# Borneol, a Bicyclic Monoterpene Alcohol, Reduces Nociceptive Behavior and Inflammatory Response in Mice

**DOI:** 10.1155/2013/808460

**Published:** 2013-04-18

**Authors:** Jackson Roberto Guedes da Silva Almeida, Grasielly Rocha Souza, Juliane Cabral Silva, Sarah Raquel Gomes de Lima Saraiva, Raimundo Gonçalves de Oliveira Júnior, Jullyana de Souza Siqueira Quintans, Rosana de Souza Siqueira Barreto, Leonardo Rigoldi Bonjardim, Sócrates Cabral de Holanda Cavalcanti, Lucindo José Quintans Junior

**Affiliations:** ^1^Núcleo de Estudos e Pesquisas de Plantas Medicinais, Universidade Federal do Vale do São Francisco, 56.304-205 Petrolina, PE, Brazil; ^2^Laboratório de Farmacologia Pré-Clínica, Universidade Federal de Sergipe, 49.000-100 São Cristóvão, SE, Brazil; ^3^Departamento de Farmácia, Universidade Federal de Sergipe,49.000-100 São Cristóvão, SE, Brazil

## Abstract

Borneol, a bicyclic monoterpene, has been evaluated for antinociceptive and anti-inflammatory activities. Antinociceptive and anti-inflammatory activities were studied by measuring nociception by acetic acid, formalin, hot plate, and grip strength tests, while inflammation was prompted by carrageenan-induced peritonitis. The rotarod test was used to evaluate motor coordination. Borneol produced a significant (*P* < 0.01) reduction of the nociceptive behavior at the early and late phases of paw licking and reduced the writhing reflex in mice (formalin and writhing tests, resp.). When the hot plate test was conducted, borneol (in higher dose) produced an inhibition (*P* < 0.05) of the nociceptive behavior. Such results were unlikely to be provoked by motor abnormality. Additionally, borneol-treated mice reduced the carrageenan-induced leukocytes migration to the peritoneal cavity. Together, our results suggest that borneol possess significant central and peripheral antinociceptive activity; it has also anti-inflammatory activity. In addition, borneol did not impair motor coordination.

## 1. Introduction

Monoterpenes, belonging to a large and diverse group of chemical compounds named “terpenes,” represent a group of naturally occurring organic compounds whose basic structure consists of two linked isoprene units, which are formed by a 5-carbon base (C_5_) each. They are the most representative molecules constituting 90% of the essential oils and have a great variety of structures [1], with several pharmacological properties assigned to them, including cardiovascular, antioxidant, anti-inflammatory, and analgesic [2, 3].

Borneol (C_10_H_18_O), a bicyclic monoterpenoid alcohol, one of the valuable medical material, senior aromatic spices, and chemical materials, has been used in food and also folk medicine in China and India. According to the Pharmacopoeia of China (2005), borneol is an important ingredient among about 63 herbal products [4]. This compound is considered a GRAS (generally regarded as safe) approved by the FDA (US Food and Drug Administration) as food flavor [5]. Additionally, borneol is a fragrance ingredient used in decorative cosmetics, fine fragrances, shampoos, toilet soaps, and other toiletries as well as in noncosmetic products such as household cleaners and detergents. Its use worldwide is in the region of 10–100 metric tonnes per annum [6]. 

Previous studies have shown that borneol has vasorelaxant effect on rat thoracic aorta [7] and neuroprotective effects [8]. Despite being inserted in pharmaceutical preparations to treat painful and inflammatory conditions, few studies have been found investigating the specific role of borneol in this regard. So, we investigated the possible antinociceptive effect of borneol in rodents.

## 2. Material and Methods

### 2.1. Animals

Male adult albino Swiss mice (25–35 g) were used throughout this study. The animals were randomly housed in appropriate cages at 22 ± 2°C on a 12 h light/dark cycle with free access to food and water. They were used in groups of six animals each. Experimental protocols and procedures were approved by the Universidade Federal do Vale do São Francisco Animal Care and Use Committee by number 024240408.

### 2.2. Acetic-Acid-Writhing-Induced Nociception

This test was performed using the method described by Koster et al. [9]. Mice were divided into six groups of six mice each. Acetic acid (0.9% v/v) was administered i.p. in a volume of 0.1 mL/10 g. Vehicle (saline), morphine (10 mg/kg), indomethacin (INDO 20 mg/kg), and borneol (BOR 5, 25, and 50 mg/kg) were administered i.p. 30 min before the injection of acetic acid. The number of abdominal constrictions produced in each group for 5–15 min after injection was counted and compared to the response in the control group. Antinociceptive activity was calculated as the percentage inhibition of abdominal constriction.

### 2.3. Formalin-Induced Nociception

The method used was similar to that described by Hunskaar and Hole [10]. Twenty microliters of 2.5% formalin (in 0.9% saline, subplantar) was injected subcutaneously into the right hind paw of the mice. The time (in seconds) spent licking and biting the injected paw was measured as an indicator of pain response. Responses were measured for 5 min after formalin injection (first phase, neurogenic) and 15–30 min after formalin injection (second phase, inflammatory). Vehicle (saline), morphine (10 mg/kg), indomethacin (INDO 20 mg/kg), and borneol (BOR 5, 25, and 50 mg/kg) were administered i.p. 60 min before the injection of formalin. Mice were observed in the chambers with a mirror mounted on three sides to allow view of all of the paws. Antinociceptive activity was calculated as the percentage inhibition of licking time.

### 2.4. Hot Plate Test

Mice were divided into five groups of six mice each. Mice were preselected on the hot plate at 55 ± 0.5°C. Licks on the rear paws were the parameters of observation. Animals showing a reaction time (defined as the latency for licking the hind feet or jumping) greater than 20 s were discarded. The animals were then treated with vehicle (saline, 0.1 mL/10 g, i.p.), morphine (10 mg/kg, i.p.), and borneol (BOR 5, 25, and 50 mg/kg, i.p.). Latency time (in seconds) for each mouse was determined on the hot plate during a maximum period of 20 s, at intervals of 30, 60, 90, and 120 min after the administration of the vehicle, extract, and morphine [11].

### 2.5. Rotarod Test

A rotarod tread mill device (Insight, Brazil) was used for the evaluation of motor coordination [12]. Initially, 24 h before the test, mice capable of remaining on the rotarod apparatus longer than 180 s (7 rpm) were selected. Thirty minutes after the administration of either borneol (BOR 5; 25, and 50 mg/kg, i.p.), vehicle (saline/Tween 80 0.2%; control group), or diazepam (DZP; 2.5 mg/kg, i.p.), each animal was tested on the rotarod apparatus at 0.5, 1, and 2 h after treatment, and the time (s) the mice were able to remain on top of the bar was recorded for up to 180 s.

### 2.6. Grip Strength Test

Grip strength test was performed using a grip strength meter (Model EFF-305, Insight, Ribeirão Preto-SP, Brazil) as previously described [13]. The grip strength meter consists of a force transducer with digital display and a metal plate with a trapeze. Each mouse was placed on the plate and was pulled by its tail with increasing force until it was unable to grasp the trapeze and the grip was broken. The instrument digitally captures and displays the peak pull-force achieved. Muscle strength was defined as the peak weight (g) indicated on the display. The value was determined individually as the mean of three trials and presented as group mean ± SEM. Mice were treated similarly of the rotarod test.

### 2.7. Leukocyte Migration to the Peritoneal Cavity

The leukocyte migration was induced by the injection of carrageenan (500 *μ*g/cavity, i.p., 500 *μ*L) into the peritoneal cavity of mice 1 h after administration of borneol (5, 25, and 50 mg/kg, i.p.) or aspirin (200 mg/kg, i.p.) by the modification of the technique previously described by Bastos et al. [14] and Leite et al. [15]. The animals (*n* = 6, per group) were euthanized by cervical dislocation 4 h after carrageenan injection. Shortly after, phosphate buffered saline (PBS) containing EDTA (1 mM, i.p., 10 mL) was injected. Immediately, a brief massage was done for further fluid collection, which was centrifuged (2000 rpm, 5 min) at room temperature. The supernatant was disposed, and 1 mL of PBS was introduced to the precipitate. An aliquot of 10 *μ*L from this suspension was dissolved in 200 *μ*L of Turk solution, and the total cells were counted in a Neubauer chamber, under optic microscopy. The results were expressed as the number of leukocytes/mL. The  percentage  of  the  leukocyte  inhibition = (1 − *T*/*C*) × 100, where *T* represents the treated groups leukocyte counts and *C* represents the control group leukocyte counts [15].

### 2.8. Statistical Analysis

 All data obtained were evaluated by one-way analysis of variance (ANOVA) followed by Dunnett's test. Differences were considered to be statistically significant when *P* < 0.05. All statistical analyses were done using Graph Pad Prism 5 (Graph Pad Prism Software Inc., San Diego, CA, USA). The percent of inhibition by an antinociceptive agent was determined for the following formula: % Inhibition = 100 × (control − experiment)/control [16].

## 3. Results and Discussion

**Figure 1:**
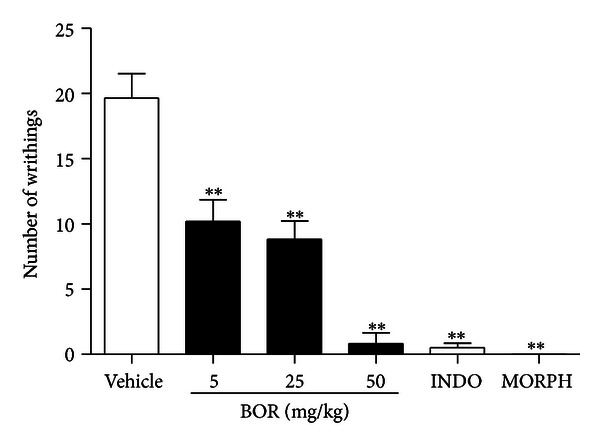
Effect of borneol (BOR), indomethacin (INDO 20 mg/kg), and morphine (MORPH 10 mg/kg) on acetic-acid-induced writhing test. Values are mean ± S.E.M. **P < 0.01, significantly different from control; ANOVA followed by Dunnett's test (*n* = 6, per group).

The present study demonstrates that borneol (5, 25 and 50 mg/kg) is capable of strongly preventing acetic-acid-writhing-induced nociception in mice when injected into intraperitoneally (i.p.) route before 30 min acetic acid administration (Figure 1). This is a classical model widely used to screen new agents with analgesic profile where both neurogenic and/or inflammatory pain is involved [17]. In acetic-acid-induced abdominal writhing, pain is elicited by the injection of an irritant such as acetic acid into the peritoneal cavity which produces episodes of characteristic stretching (writhing) movements, and inhibition of the number of episodes by analgesics is easily quantifiable [9]. Drugs-like analgesics can inhibit this nociceptive behavior [12]. 

**Figure 2:**
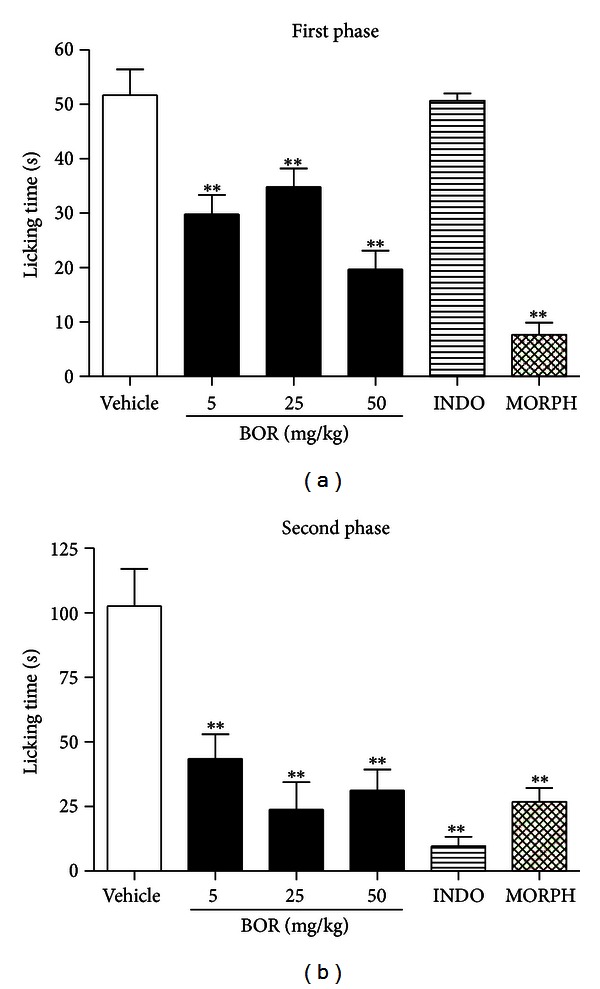
Effect of borneol (BOR), indomethacin (INDO 20 mg/kg), and morphine (MORPH 10 mg/kg) on first phase (a) and second phase (b) of the formalin test. Values are mean ± S.E.M.; **P < 0.01, significantly different from control; ANOVA followed by Dunnett's test (*n* = 6, per group).

The systemic administration of borneol, all doses, significantly reduced (*P* < 0.01) pain behavior caused by formalin injection in both phases of the formalin test (Figures 2(a) and 2(b)). This test is a very useful method for not only assessing antinociceptive drugs but also helping in the elucidation of the action mechanism [18]. The subcutaneous injection of formalin in the mice paw induces a biphasic response. The neurogenic phase (first phase) is probably a direct result of stimulation in the paw and reflects centrally mediated pain with release of substance P, while the late phase (second phase) is due to the release of histamine, serotonin, bradykinin, and prostaglandins [17].

Since acute treatment with borneol elicited a significantly reduction of painful behavior induced by formalin (in the first phase), this result indicates a possible neurogenic (central) component in analgesic profile of monoterpene. Thus, we conducted a hot plate test to evaluate possible involvement of supraspinal component in this response. This test consists of introducing a mouse into an open-ended cylindrical space with a floor consisting of a metallic plate that is heated by a thermode or a boiling liquid [19]. A plate heated to a constant temperature produces two behavioral components that can be measured in terms of their reaction times, namely, paw licking and jumping. Both are considered to be supraspinally integrated responses [17]. The borneol-treated mice lack reduces the painful behavior only in higher dose and at time 0.5 or 2 h after treatment (Table 1). This inconsistent response may have occurred due to a lack effect of borneol on opioid system; after all, the hot plate test is more useful to evaluate opioids drugs [17]. Additionally, this results support the hypothesis that many monoterpenes do not exhibit dose-dependent effects [3]; thereby, it is necessary to find the most appropriate dose range that shows effectiveness. 

Anyway, according to Granger et al. [20], while many possible mechanisms for the actions of sedative borneol have been proposed, these monoterpenes have been primarily linked, in electrophysiology studies, with functions associated with the neurotransmitter GABA_A_. Additionally, Quintans-Júnior et al. [21] reinforce this effect demonstrating that borneol possess anticonvulsant and sedative properties due to the modulation of the GABA system. Several studies demonstrate the hole of GABA system in pain modulation [22, 23]. Thus, we do not disregard the possibility that the antinociceptive profile of intraperitoneal injected borneol might involve, at least, others central nervous system (CNS) effects like anticonvulsant or anxiolytic activities and a possible involvement of GABA system.

**Figure 3:**
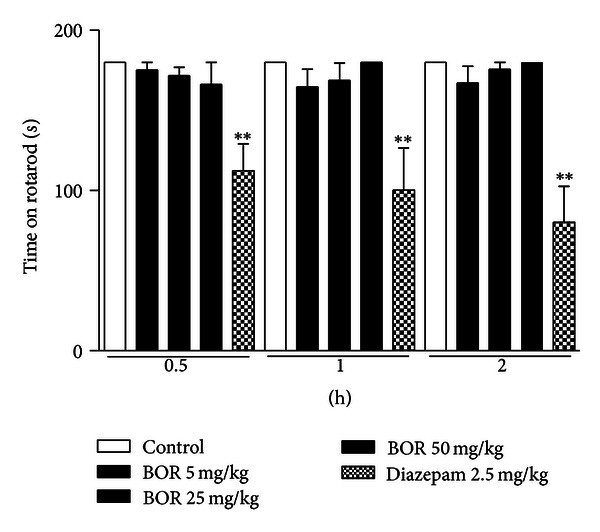
Effect of borneol (BOR) and diazepam on rotarod test. Values are mean ± S.E.M.; **P < 0.01, significantly different from control; ANOVA followed by Dunnett's test (*n* = 6, per group).

**Figure 4:**
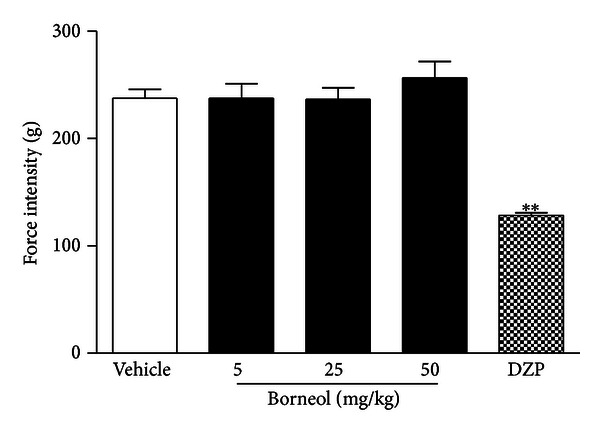
Effect of borneol (BOR) and diazepam (2.5 mg/kg) on grip strength test. Values are mean ± SEM.; **P < 0.01, significantly different from control; ANOVA followed Dunnett's test (*n* = 6, per group).

On the other hand, in order to clarify if the pharmacological effects of borneol would be consequent to a central activity interference on motor function or motor coordination, the activity of borneol was also evaluated on rotarod and grip strength meter apparatus that are classical models for screening CNS or myorelaxant actions providing information about psychomotor performance. As shown in Figures 3 and 4 borneol-treated mice did not cause any motor disturbance on rotarod and grip strength meter test. This effect corroborates that analgesic profile demonstrated by borneol is a direct action in modulate pain by an understanding mechanism.

**Figure 5:**
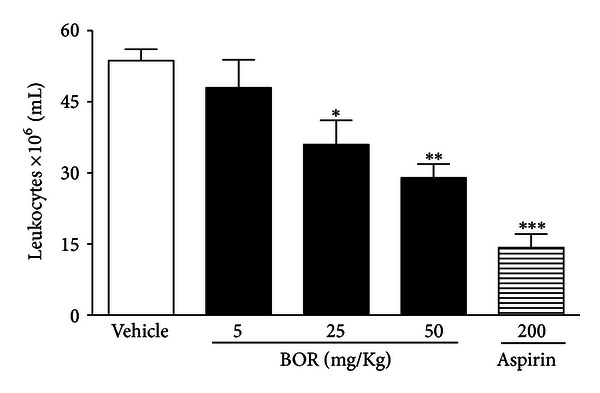
Effect of borneol (BOR) and aspirin on leukocyte migration into the peritoneal cavity induced by carrageenan in mice. Groups of rats were pretreated with vehicle (control group), aspirin (200 mg/kg, p.o.), or BOR (5, 25, or 50 mg/kg, i.p.) 60 min before carrageenan (500 *μ*g/cavity, 500 *μ*L, i.p.)-induced peritonitis. Cell counts were performed at the time 4 h after the injection of carrageenan. Each value represents the mean ± S.E.M. Asterisks denote statistical significance, *P < 0.5, **P < 0.01, or ***P < 0.001 related to control group. ANOVA followed by Dunnett's test (*n* = 6, per group).

Since borneol produced a marked analgesic effect in acetic acid writhing-induced nociception and in later phase of formalin test, such that this phase has a strong participation of inflammatory mediators, we investigate the possible effect of borneol on leukocytes migration. Cell recruitment during inflammation depends on the orchestrated release of local mediators that are responsible for local vascular and tissue changes as well as for the recruitment of host defense cells [24]. The inflammation induced by carrageenan involves cell migration, plasma exudation, and production of mediators, such as nitric oxide, prostaglandin E_2_, interleukin (IL)-1*β*, IL-6, and tumor necrosis factor (TNF-*α*) [25]. Borneol, in higher doses, reduced leukocyte migration induced by i.p. injection of inflammatory agent (carrageenan) in peritonitis model (Figure 5).

## 4. Conclusions

In summary, it can be concluded that borneol is endowed with peripheral and centrally acting analgesic properties (without producing motor deficit) as well as anti-inflammatory profile. However, a more in-depth evaluation of the mechanisms involved should be performed. Our results also support that borneol has a therapeutic potential for painful and inflammatory disorders.

## Figures and Tables

**Table 1:** Effect of borneol and morphine on hot plate test in mice.

Groups	Dose (mg/kg)	Latency time (s)			
		0.5 h	1 h	1.5 h	2 h
Vehicle	—	4.43 ± 0.48	7.52 ± 0.74	6.07 ± 0.70	6.52 ± 0.76
Borneol	5	7.07 ± 0.42	9.77 ± 1.28	8.48 ± 0.83	7.47 ± 0.41
	25	8.16 ± 0.89	7.58 ± 0.73	9.79 ± 0.48	8.27 ± 0.59
	50	9.86 ± 0.83*	9.18 ± 0.65	8.89 ± 0.91	9.84 ± 0.92*
Morphine	10	13.79 ± 2.52**	12.19 ± 1.67*	12.34 ± 1.75**	12.80 ± 1.17**

Values are mean ± SEM, *n* = 6; *P < 0.05; **P < 0.01 significantly different from control (ANOVA followed by Dunnett's test).
